# Repetitive brief ischemia accelerates tibial shaft fracture healing: a 5-years prospective preliminary clinical trial (PCT)

**DOI:** 10.1186/s12891-021-04515-y

**Published:** 2021-07-20

**Authors:** Dong Wang, Yang Liu, Wenrui Lv, Wei Liang, Xiaobin Zhou, Yin Ding, Junlin Zhou

**Affiliations:** 1 Department of Orthopedics, Beijing Chaoyang Hospital, Capital Medical University, 8 Gongren Tiyuchang Nanlu, Chaoyang District, Beijing, 100020 China; 2 Department of Respiratory and Critical Care Medicine, Beijing Chaoyang Hospital, Capital Medical University, 8 Gongren Tiyuchang Nanlu, Chaoyang District, Beijing, 100020 China; 3 Beijing Tongzhou Xinhua Hospital, Beijing, 101100 China; 4 Third Department of Traumatology, The Third Hospital of Shijiazhuang, Shijiazhuang, 050000 China

**Keywords:** Repetitive brief ischemia, Tibia shaft fractures, Bone healing, Intermedullary nail, Prospective clinical trials, Serum vytokines

## Abstract

**Background:**

This study was to evaluate the effects of repetitive brief ischemia (RBI) on bone healing in patients with tibial shaft fractures.

**Methods:**

In this prospective clinical trial, patients with tibia shaft fractures were enrolled between January 2016 and January 2021. The intermittent pneumatic compression (IPC) device was used to make RBI on the affected limb after surgical operation 24 h. The inflation pressure was the systolic pressure of patients + 50 mmHg. Patients were received 30 s inflation/30 s deflation 30 times twice a day for 4 weeks. The primary outcome was bone healing time and the secondary outcomes were the rates of delayed union and nonunion, the rates of venous thrombosis of lower limbs, Johner-Wruhs scores, Lysholm knee score, VAS scores, postoperative complications, serum BMP-2, osteocalcin (OC) and bone specific alkaline phosphatase (BS-ALP).

**Results:**

A total of 32 patients were enrolled finally and all were completed with a 12 months follow-up. All the fractures were healed and the bone healing time was 3(1) months in RBI group. However, the bone healing time of control group was 4(1) and there was statistical difference between the two groups (*p* < 0.01). No patient with fracture delayed union, nonunion and venous thrombosis of lower limbs in RBI group. 2 patients were delayed union in the control group. In RBI group, the Lysholm knee score was 83(6) at 6 months and 100(8) at 12 months. In the control group, the score was 78(4) and 90.5(17), there was statistical difference between the two groups (*p* < 0.01, *p* = 0.014, respectively). VAS scores were postoperative 2 weeks 6(1) in RBI group and 7(0.5) in the control group, there was statistical difference between the two groups (*p* = 0.016). There were 2 patients with intramuscular venous thrombosis of lower extremity in control group. Besides, RBI treatment increased the serum BMP-2, OC and BS-ALP at postoperative 2 weeks and 1 month.

**Conclusions:**

RBI is a new method to accelerate bone healing in tibia shaft fracture patients and is a simple and noninvasive method.

**Trial registration:**

Chinese clinical trial registry, ChiCTR-INR-17014208. Registered 28 December 2017—Retrospectively registered.

**Supplementary Information:**

The online version contains supplementary material available at 10.1186/s12891-021-04515-y.

## Introduction

Fracture is the most common trauma in orthopaedics and causes more than 150,000 patients hospitalisations in Australia each year [1]. Although there are many ways to promote bone healing and some of them have been used in clinic, the rate of delayed union or nonunion has not decreased significantly [2, 3]. The rate of bone nonunion is about 5–10% and tibia fracture nonunion or delayed union is 25% [3], besides, the direct costs for healthcare and loss of productivity in the first 6 months post-injury are about $23,000 for each limb fracture. Once nonunion occurs, most of patients will undergo a revision surgery, which will cause a secondary injury of the patients. Even if the fracture is healed after revision treatment, it is difficult to recover the function of the affected limb. Therefore, as the nonunion rate of tibial fracture, we are looking for a new method to accelerate bone healing and reduce the nonunion rate.

Ischemia preconditioning can improve skin flap survival which gives us a new idea. In 2016, we explored the effects of affected limb repetitive brief ischemia (RBI) on bone healing in a rat tibia fracture model and found that RBI treatment can promote fracture healing and stimulate the secretion of IGF-2 [4]. In 2019, we further discussed that whether health limb RBI can accelerate fracture healing and raised that RBI on the affected side or the healthy side limb could promote bone healing and boosted the synthesis of BMP-2, VEGF, TGF-β, and ALP in the fracture region [5].

At present, there is no clinical study on the effect of RBI on fracture healing. This purpose of this study was to analyze the effects of RBI on bone healing in patients with tibial shaft fractures and preliminarily discuss the mechanism.

## Methods

### Trial design

This study was a 5-years prospective clinical trial of RBI on tibial shaft fracture healing and registrated in Chinese clinical trial registry (registration number was ChiCTR-INR-17014208, 28/12/2017). Primary outcome was the bone healing time, in addition, the secondary outcomes were the rates of delayed union and nonunion, the rates of venous thrombosis of lower limbs, Johner-Wruhs scores, Lysholm knee score, visual analogue scale (VAS) scores, postoperative complications, such as wound infection, fracture of internal fixation, loss of fracture reduction and vascular or nerve injury, and serum cytokines, such as bone morphogenetic protein-2 (BMP-2), osteocalcin (OC) and bone specific alkaline phosphatase (BS-ALP). The study was performed at Beijing Chao-Yang hospital affiliated to Capital Medical University and the third hospital of Shijiazhuang between January 2016 and January 2021. It was approved by the ethics committee of Beijing Chao-Yang Hospital Affiliated to Capital Medical University (2017–5–8–2) and the third hospital of Shijiazhuang (2021–018). We could not reach the sample size required for the registration of this clinical trial even if the study time was extended. Therefore, this study was converted from a randomized controlled trial to a small size prospective clinical trial.

### Sample size

Stata 12.0 software was used to calculate the sample size. There is no relevant clinical study on the effects of RBI on fracture healing and only a few animal studies clarified that RBI accelerated bone healing. Therefore, the relevant pre-experiment was carried out and two patients were enrolled. The fracture healing time of RBI therapy group were 2.5, 3.0 months. Besides, the fracture healing time of tibia shaft fracture was 4.2 ± 1.025 months as described by Lu Y [6]. Alpha was set as 0.01 (two-sided) and power was set as 0.95. A total of 26 patients were required. The ratio of RBI therapy group and control group was 1.

### Participants

The inclusion criteria for participants was as follows: patients aged from 18 to 65 years old; patients with fresh, closed AO/OTA type 42 tibial shaft fracture; patients treated with closed and interlocking intramedullary nail internal fixation (the same operation team); patients signed informed consent for clinical research.

The exclusion criteria for participants was as follows: patients with pathological fractures and open fractures; patients combined with other fractures; patients with vascular diseases, such as aneurysm, arterial embolism, thromboangitis obliterans, arteriosclerotic obliterans, polyarteritis and deep venous thrombosis; patients with metabolic and immune diseases, such as systemic lupus erythematosus, polymyositis and Sjogren’s syndrome; patients treated with long-term glucocorticoid therapy; patients with partial skin lesions at the root of the affected thigh and not suitable for RBI treatment; patients not cooperated with the study, withdrawal and loss of visit; patients who did not meet the inclusion criteria.

Two clinical observers (YL and DW) with clinical research experience enrolled the patients strictly according to the inclusion criteria. The baseline information of participants were recorded, such as patient’s name, gender, age, affected side, body mass index (BMI), the cause of bone fracture, closed or opened fracture, combination diseases (hypertension, diabetes mellitus, coronary heart disease and cerebral infarction), smoking or not, excessive drinking or not, the time of trauma to operation, fracture classification (AO/OTA).

### Interventions

**Fig. 1:**
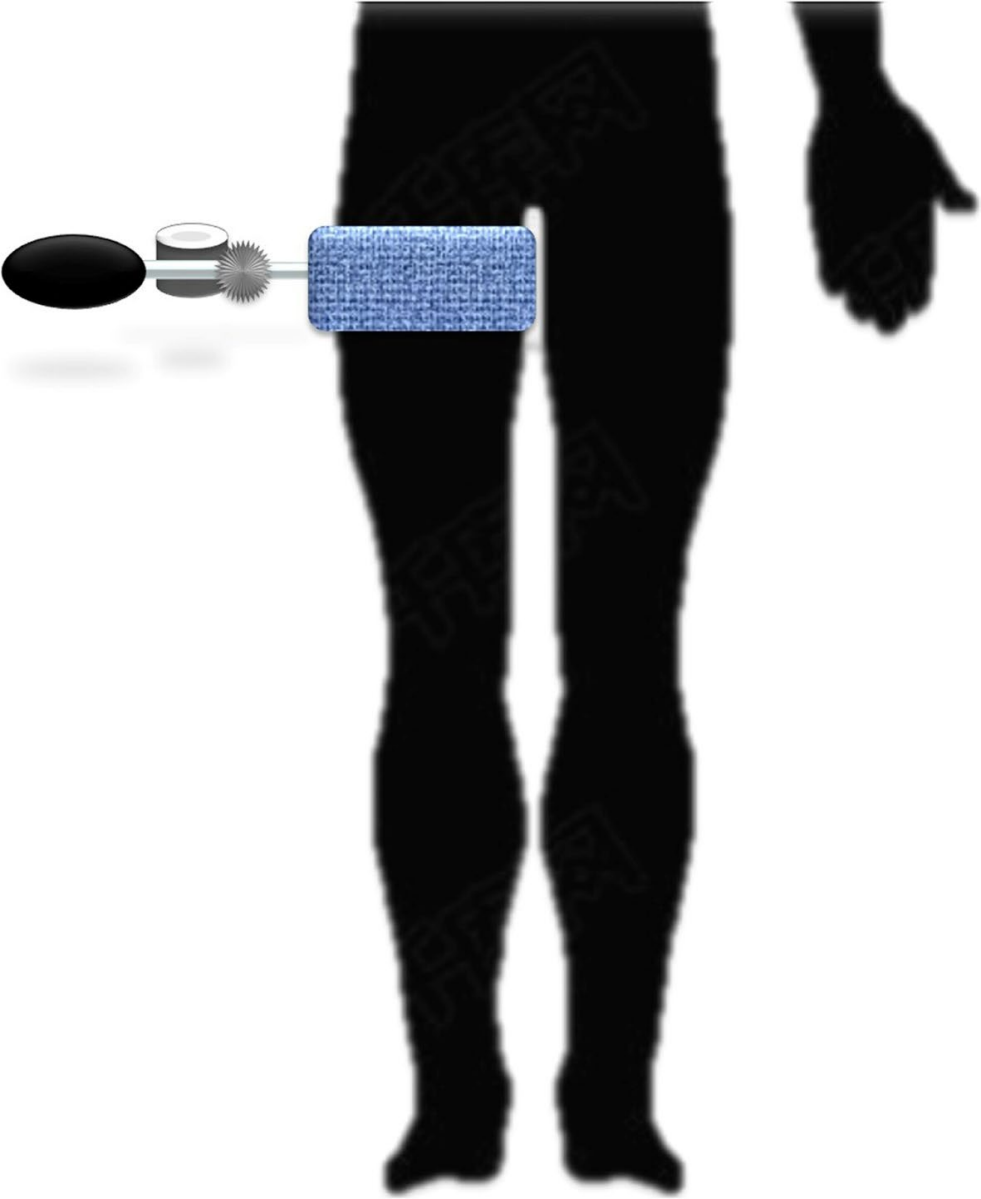
The diagrammatic drawing of RBI using

Participants received RBI treatment after surgical operation 24 h. The intermittent pneumatic compression (IPC) device (Zosing, Henan, China) was used to make RBI. The IPC device was described in previous studies [7–9] (Fig. 1). Briefly, it contained two parts: the air-bag part and cuff part. The air bag part included air-bag, pressure gauge and valve. The cuff part included a connecting tube and an inflatable cuff. The inflatable cuff was placed at the thigh root of the affected limb and inflated by squeezing the air-bag. The pressure of cuff was the systolic pressure of patients + 50 mmHg. After inflation for 30 s (or terminated at any time if the patient’s intolerance), the valve was opened and the gas in the cuff was discharged until the pressure to 0 mmHg. After deflation for 30 s, the valve was closed and cuff was inflated again. The above inflation/deflation cycle was repeated for 30 times twice a day for 4 weeks by two experienced researchers (WRL and WL). The researchers guided and recorded the patients through video dialogue after the patients discharged from hospital.

The patients in the control group did not receive the RBI therapy.

### Outcomes

Two experienced researchers (DW and XBZ) recorded the outcomes. The primary outcome was the bone healing time and bone fracture healing criteria were no pressure pain and longitudinal percussion pain in the fracture area, fuzzy fracture line on X-ray, 3/4 cortical connection and no pain after complete weight-bearing [10, 11]. The secondary outcomes were the rates of delayed union and nonunion, the rates of venous thrombosis of lower limbs, Johner-Wruhs scores, Lysholm knee score, VAS scores, postoperative complications, and serum BMP-2, OC and BS-ALP [12, 13].

Delayed union criteria were that the fracture had not reached the healing criteria after 4 months [14, 15]. There was less callus, slightly decalcified, obvious fracture line in the fracture area by X-ray, but no osteosclerosis was at the end of fracture [14, 15]. The nonunion of fracture was that the fracture had not researched healing criteria after 8 months or the fracture end had been sclerotic [14, 15].

The postoperative outpatient follow-up timepoints were 1, 2, 3, 4, 6, 8, 12 months. The end point of the trial was postoperative 12 months or patients diagnosed as nonunion. X-rays was used to evaluate the fracture healing at postoperative 1, 2, 3, 4, 6, 8, 12 months. Doppler ultrasound of lower extremity veins was used to diagnosed venous thrombosis of lower limbs at postoperative 1, 2, 3, 6, 12 months. Two researchers (DW and XBZ) were recorded the Johner-Wruhs scores, Lysholm knee score at postoperative 6, 12 months, in addition, also recorded VAS scores at preoperative and postoperative 2 weeks, 1, 2, 3 months. Fasting blood was collected from the patients preoperative 1 day, postoperative 2 weeks, 1, 2, 3 months. The serum BMP-2, OC and BS-ALP were detected by ELISA.

### Laboratory methods

Human bone morphogenetic protein 2 ELISA kit (CSB-E04507h, Cusabio, China), human osteocalcin ELISA kit (CSB-E05128h, Cusabio, China) and human bone alkaline phosphatase ELISA kit (CSB-E09033h, Cusabio, China) were used. 2 ml blood collected from patients was centrifuged at 2500 rm/r, 25 min. Then, BMP-2, OC and BS-ALP of supernatant were detected according to the instructions of ELISA kit.

### Statistical methods

Stata 12.0 software was used to analyzed the data. Kolmogorov-Smirnova test was used to evaluate the normality of the measurement data. If the measurement data followed normal distribution, it was described as means ± standard and compared by independent-sample t test. If the measurement data obeyed the skewed distribution, it was represented by median (quartile range) and compared by Mann–Whitney U test. The enumeration data was represented by occurrence rate and compared by Chi-square test or Fischer’s exact test. *P* < 0.05 was considered as statistical difference.

## Results

### Study population

A total of 32 patients were enrolled and all were completed with a 12 months follow-up. Each group was 16 patients, 11 were male and 5 were female. The age was 47.75 ± 15.28 in RBI group and 39.75 ± 15.08 in control group. The fracture type in each group was: 11 patients were 42A1, 1 patient was 42A2, 3 patients were 42B2 and 1 patient was 42C2. The baselines of participants were in Table 1 (the detail of each patient was in supplementary materials). There was no significant difference in baseline data between the two groups, and the two groups were comparable.

**Table 1:** The baselines of participants

	RBI therapy group (*n* = 16)	Control group (*n* = 16)	*P* value
Age(y)	47.75 ± 15.28	39.75 ± 15.08	0.147
Sex			
Female	5	5	< 0.99
Male	11	11	< 0.99
BMI(kg/m2)	23.25 ± 2.80	26.01 ± 5.22	0.073
Affected side			
Left	8	10	0.722
Right	8	6	0.722
Fracture type			
42A1	11	11	< 0.99
42A2	1	1	< 0.99
42B2	3	3	< 0.99
42C2	1	1	< 0.99
Injury reason			
Falling by self	13	9	0.252
Traffic accident	3	7	0.252
Timing between trauma and surgical treatment(d)^a^	4.0 (6.0–2.0)	5.0 (3.5)	0.761
Combination diseases			
Hypertension	6	2	0.22
Diabetes mellitus	3	0	0.226
Coronary heart disease	2	0	0.484
Cerebral infarction	0	0	< 0.99
Allergic history	3	1	0.6
Smoking history	8	2	0.054
Excessive drinking history	4	3	< 0.99

^a^Because the data is skewed distribution, it was represented by median (quartile range) and compared by Mann–Whitney U test. The enumeration data was compared by Fischer’s exact test as the small size

### Primary outcome

Two experienced researchers recorded the outcomes. All the fractures were healed and the bone healing time was 3(1) months in RBI group. However, the bone healing time of control group was 4(1) and there was statistical difference between the two groups (*p* < 0.01) (Table 2, the detail of each patient was in supplementary materials).

**Table 2:** The outcomes of participants

	RBI therapy group (*n* = 16)	Control group (*n* = 16)	*P* value
Operation time(h)	2.29 ± 0.74	1.96 ± 0.75	0.217
Intraoperative blood loss (ml)^a^	100 (50)	50 (40)	0.055
Bone healing time(m)^a^	3 (1)	4 (1)	< 0.01
Delayed union and nonunion(n)	0	2	0.484
Venous thrombosis of lower limbs(n)	0	3	0.226
Johner-Wruhs scores^a^(%)			
6 months	100%	87.50%	0.484
12 months	100%	87.50%	0.484
Lysholm knee score^a^(s)			
6 months	83 (6)	78 (4)	< 0.01
12 months	100 (8)	90.5 (17)	0.014
VAS scores^a^(s)			
Preoperative	8 (1)	7.5 (1)	> 0.99
2 weeks	6 (1)	7 (0.5)	0.016
1 month	4 (1)	4 (1.5)	0.187
2 months	3 (1.5)	4 (1)	0.146
3 months	3 (0.5)	3 (2)	0.84
Postoperative complications(n)	0	3	0.226

^a^Because the data is skewed distribution, it was represented by median (quartile range) and compared by Mann–Whitney U test. The enumeration data was compared by Fischer’s exact test as the small size. Johner-Wruhs scores was shown by excellent and good rate, besides, all the postoperative complications were venous thrombosis

### Secondary outcomes

There was no patient with fracture delayed union and nonunion and also no patient with venous thrombosis of lower limbs in RBI group. 2 patients were delayed union and no patient was nonunion in the control group. In RBI group, the Lysholm knee score was 83(6) at 6 months and 100(8) at 12 months. In the control group, the Lysholm knee score was 78(4) at 6 months and 90.5(17) at 12 months, there was statistical difference between the two groups (*p* < 0.01, *p* = 0.014, respectively). VAS scores were preoperative postoperative 2 weeks 6(1) in RBI group and 7(0.5) in the control group, there was statistical difference between the two groups (*p* = 0.016). There were 2 patients with postoperative complications, all were intramuscular venous thrombosis of lower extremity, however, there was no statistical difference between the two groups (*p* = 0.226) (Table 2, the detail of each patient was in supplementary materials).

**Fig. 2:**
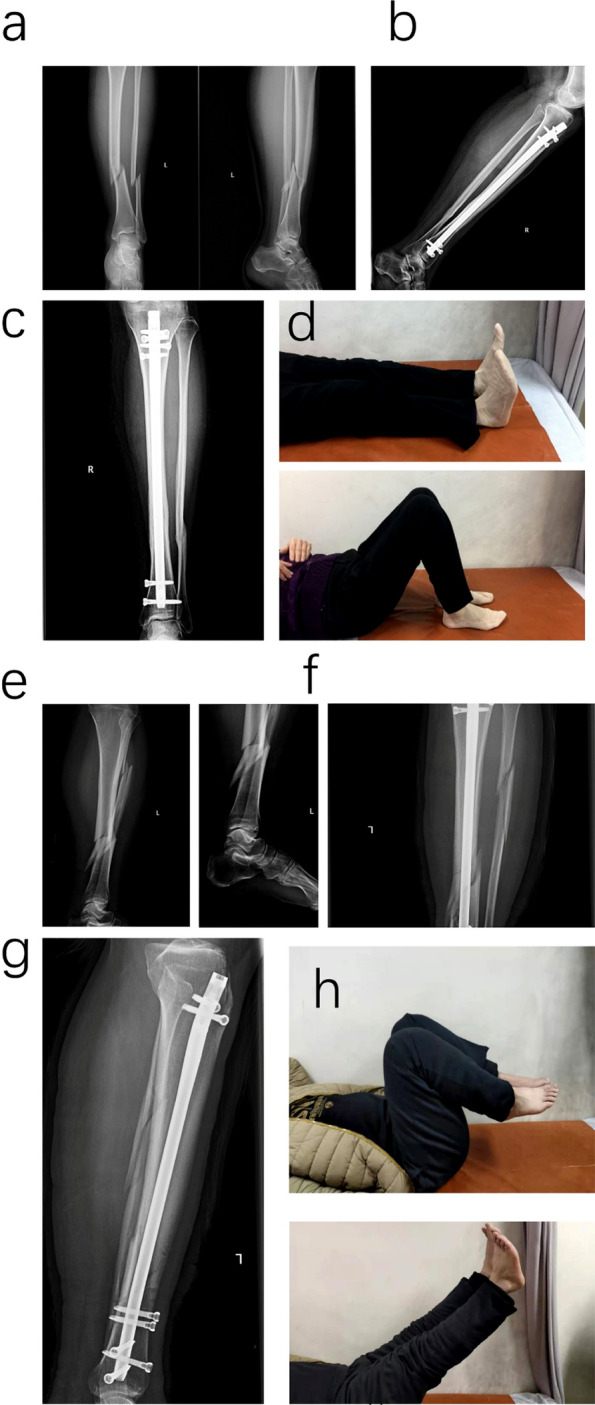
X-rays and functional activity of the RBI group. **a**-**d** Series number 14 patients. **a** Preoperative X-rays showed tibia shaft fracture and fracture type was 42A1; **b** and **c** X-rays of postoperative 3 months showed bone healed. **d** the limb activity picture showed the satisfied limb motion. **e**–**h** Series number 1 patients. **e** Preoperative X-rays showed tibia shaft fracture and fracture type was 42C2; **f**–**h** X-rays of postoperative 2 months showed that fracture was not healed but the limb motion was satisfied

**Fig. 3:**
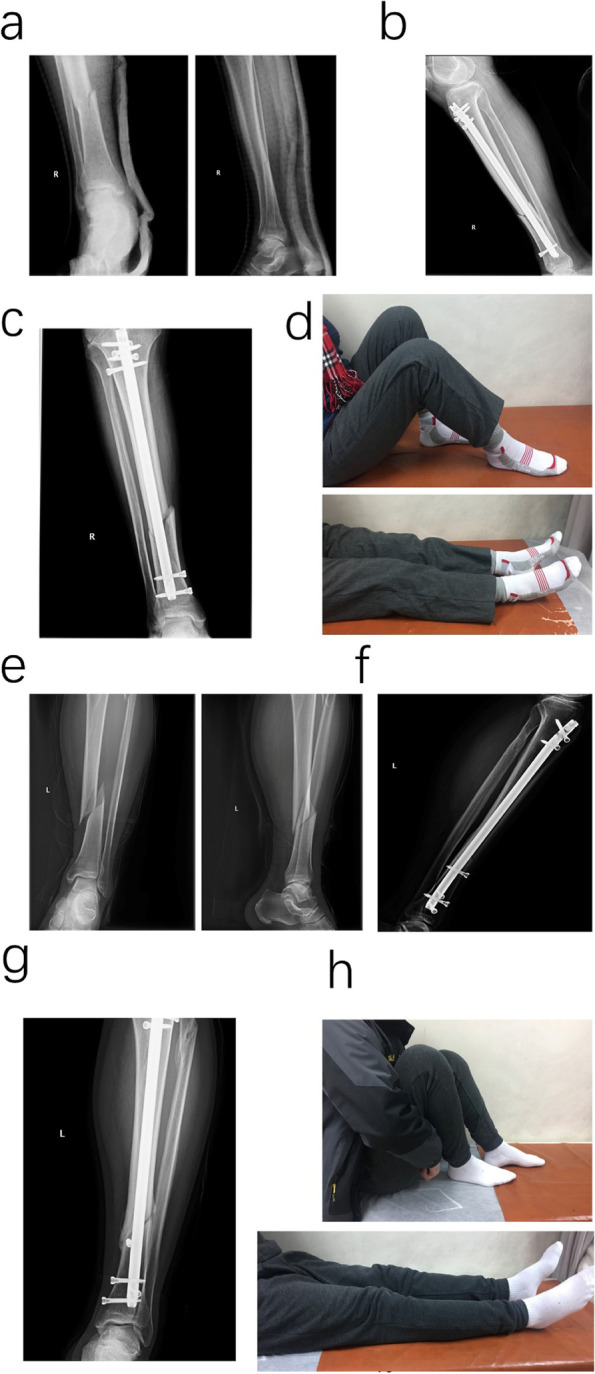
X-rays and functional activity of the control group. **a**-**d** Series number 2 patients. **a** Preoperative X-rays showed fracture type was 42A1; **b** and **c** X-rays of postoperative 2 months showed no obvious callus formation. **d** The limb activity picture showed the limb motion. **e**–**h** Series number 4 patients. **e** Preoperative X-rays showed fracture type was 42A1; **f**–**h** X-rays of postoperative 3 months showed that the fracture line could still be seen although the callus formed, besides, the limb motion was not recovery

The serum BMP-2 were postoperative 2 weeks 224.69 ± 15.17 pg/ml, 1 month 211.93 ± 16.79 pg/ml in RBI group and 206.86 ± 12.49 pg/ml, 200.71 ± 10.23 pg/ml in the control group, there was statistical difference between the two groups (*p* < 0.01, *p* = 0.031, respectively). The serum OC were postoperative 2 weeks 25.31 ± 0.62 ng/ml, 1 month 22.97 ± 0.55 ng/ml in RBI group and 24.61 ± 0.33 ng/ml, 22.19 ± 1.27 ng/ml in the control group, there was statistical difference between the two groups (*p* < 0.01, *p* = 0.037, respectively). The serum BS-ALP were postoperative 2 weeks 124.81 ± 7.98 ng/ml, 1 month 122.60 ± 8.53 ng/ml in RBI group and 118.91 ± 4.52 pg/ml, 110.07 ± 4.14 pg/ml in the control group, there was statistical difference between the two groups (*p* = 0.017, *p* =  < 0.01, respectively) (Table 3, the detail of each patient was in supplementary materials) (Figs. 2 and 3).

**Table 3:** The serum cytokines of participants

	RBI therapy group (*n* = 16)	Control group (*n* = 16)	*P* value
BMP-2(pg/ml)			
Preoperative	191.75 ± 13.32	194.86 ± 11.94	0.492
2 weeks	224.69 ± 15.17	206.86 ± 12.49	0.001
1 month	211.93 ± 16.79	200.71 ± 10.23	0.031
2 months	185.26 ± 15.80	187.63 ± 11.30	0.63
3 months	178.15 ± 14.10	173.99 ± 9.55	0.337
OC(ng/ml)			
Preoperative	23.26 ± 0.40	23.03 ± 0.56	0.184
2 weeks	25.31 ± 0.62	24.61 ± 0.33	0.001
1 month	22.97 ± 0.55	22.19 ± 1.27	0.037
2 months	19.63 ± 0.31	19.16 ± 0.92	0.07
3 months	17.28 ± 0.41	16.95 ± 0.60	0.086
BS-ALP(ng/ml)			
Preoperative	104.47 ± 9.03	110.40 ± 8.34	0.063
2 weeks	124.81 ± 7.98	118.91 ± 4.52	0.017
1 month	122.60 ± 8.53	110.07 ± 4.14	< 0.001
2 months	105.94 ± 5.86	102.06 ± 5.95	0.073
3 months	99.18 ± 5.64	98.63 ± 6.07	0.792

## Discussion

Tibia shaft fracture is a common fracture type in clinic and has a 24% incidence rate in work-age adults [1–3, 16]. The nonunion rate of tibia shaft fracture is about 14% that is much higher than the average nonunion rate of long-bone fracture [1–3]. As for reducing disruption of local blood supply, intramedullary nails have been wildly used in clinic at present [17–19]. Despite strict adherence to the guidelines, the healing time and nonunion rate of tibial shaft fractures are not decreased significantly [1–3].

This study was to explore a method to promote bone healing. RBI was a method to accelerate endogenous promoting mechanism. The previous animal studies showed that RBI could obviously promote bone healing [4, 5]. We established a rat tibia shaft fracture model and used 0.8 mm K-wire as an intermedullary fixator. IPC of rats was used to make affected side or health side limb RBI. We found that RBI accelerated bone healing both doing on the affected side and health side limb. Besides, RBI increased the expression of serum and bone fracture site BMP-2, vascular endothelial growth factor (VEGF), ALP and transforming growth factor-β1 (TGF-β1). RBI on the health side inhibited the secretion of interleukin 6 (IL-6) and promoted the secretion of interleukin 10 (IL-10). However, RBI on the affected side limb decreased the level of IL-10 and accelerated the IL-6 synthesis [5].

Based on the animal experiment finding, we tried to use RBI on tibia shaft fracture patients. The study is a prospective clinical trial and has the strict inclusion criteria. We enrolled patients aged from 18 to 65 years and with fresh, closed fracture, which excluded the influences of children, open fractures and old fractures. The participants were received a similar surgical operation and we excluded the patients received plate fixation. Meanwhile, the operation was carried out by the same team. By these limitations, the impacts of operation on outcome were minimized. RBI made the limb about 30 s ischemia and cause a change of blood flow in patients, so we excluded the patients with vascular diseases, especial those with venous thrombosis. Immune diseases and long-term glucocorticoid therapy have an impact on fracture healing, so patients with these disease or drug history were excluded.

Finally, 32 patients were enrolled and each group was 16 patients. The age was 47.75 ± 15.28 and 13 was fallen by themselves in RBI group, besides, the age was 47.75 ± 15.28 and 9 was fallen by themselves in the control group which meant that most patients were the low energy injuries. After 4 weeks RBI, we found that the bone healing time was 3(1) months that was much shorter than previous literature reported and the control group [1–3]. In additions, there was no patient with fracture delayed union and nonunion. RBI might reduce the nonunion incidence [1–3]. The excellent-good rate of Johner-Wruhs scores was 100% that meant the limb function recovered satisfactorily. Besides, RBI decreased the VAS scores compared with previous studies and the control group at postoperative 2 weeks [17–20]. There was no venous thrombosis of lower limbs and postoperative complication occurred in any of the patients in RBI group so we thought that RBI might be safety to patients, however, this conclusion was needed many large sample-size randomized controlled trials to prove.

The limitations of this study were too many. First, the sample size of this trial was too small and clinical studies with large sample size are needed as this study was only an exploratory trial. Second, we were unable to accurately assess the effects of RBI on fracture healing as the small sample size. Thirdly, the included patients had a wide age range, both high-trauma and low-trauma injuries, so it is necessary to expand the sample size for subgrouping analysis. Finally, we did not make the blinding so there may be selection bias.

## Conclusions

RBI is a new method to accelerate bone healing in tibia shaft fracture patients and is a simple and noninvasive method. It can be widely used, especially in poor countries or economically underdeveloped areas.

## Supplementary Information

**Additional file 1.**

## Data Availability

The supplemental data used to support the findings of this study have been submitted in the supplementary materials.

## Abbreviations

RBI: Repetitive brief ischemia
IGF-2: Insulin-like growth factor-2
BMP-2: Bone morphogenetic protein-2
VEGF: Vascular endothelial growth factor
TGF-β: Transforming growth factor-β
ALP: Alkaline phosphatase
OC: Osteocalcin
BS-ALP: Bone specific alkaline phosphatase
BMI: Body mass index
IPC: Intermittent pneumatic compression
VAS: Visual analogue scale
ELISA: Enzyme-linked immuno sorbent assay
